# Revision of the Palaearctic *Gasteruption assectator* aggregate, with special reference to Sweden (Hymenoptera, Gasteruptiidae)

**DOI:** 10.3897/zookeys.615.8857

**Published:** 2016-09-07

**Authors:** Niklas Johansson, Cornelis van Achterberg

**Affiliations:** 1 Fredriksberg, Baskarp 566 92 Habo, Sweden; 2 Research Associate, Department of Terrestrial Zoology, Naturalis Biodiversity Center, Postbus 9517, 2300 RA Leiden, The Netherlands

**Keywords:** Europe, Gasteruption boreale, Gasteruption nigritarse, key, new records, re-instated species, Sweden, synonyms

## Abstract

The Palaearctic species of the *Gasteruption assectator* aggregate (Hymenoptera, Gasteruptiidae) are revised and three species are recognised. Two species are re-instated: *Gasteruption boreale* (Thomson, 1883), **stat. n.** and *Gasteruption nigritarse* (Thomson, 1883), **stat. n.**, and both are excluded from the synonymy with *Gasteruption assectator* (Linnaeus, 1758). The general distribution of both species is given for Europe and in detail for Sweden. A key to the valid Palaearctic species of the *Gasteruption assectator* aggregate is given; key characters and primary types are illustrated. Four new synonyms are listed: *Foenus fumipennis* Thomson, 1883, *Trichofoenus breviterebrae* Watanabe, 1934, and *Gasteruption margotae* Madl, 1987, are synonymized with *Gasteruption boreale* (Thomson, 1883) and *Gasteruption brevicauda* Kieffer, 1904, with *Gasteruption undulatum* (Abeille de Perrin, 1879).

## Introduction

The predator inquiline wasp *Gasteruption assectator* (Linnaeus, 1758) (Hymenoptera, Gasteruptiidae) has been considered a very common species with wide intraspecific variation concerning both morphology and colouration (e.g. van Achterberg and Talebi 2014). When working on an updated revision of the Nordic *Gasteruption* species the first author noticed that the specimens from a restricted geographical range could be clustered into three separate morphospecies. The discovery of a couple of hitherto unknown features of the females made it fairly easy to separate the morphospecies involved. Further studies of a larger number of specimens and conclusions drawn from practical field work showed that the three morphospecies have a significant difference in geographical distribution and habitat preference in Sweden. Studies of the type material of the supposed synonyms of *Gasteruption assectator* showed that *Foenus borealis* Thomson, 1883, and *Foenus nigritarsis* Thomson, 1883, are the oldest available names for these valid species. Nearctic synonyms listed by Smith (1996) are not accounted for here because their types need to be studied first. The synonymisation of *Gasteruption brevicauda* Kieffer, 1904, with *Gasteruption assectator* made by Madl (1987a) is here rejected and it is considered to be conspecific with *Gasteruption undulatum* (Abeille de Perrin, 1879), syn. n.

## Material

The first author studied specimens of the *Gasteruption assectator* aggregate deposited in the Evolutionsmuseet, Uppsala; The Swedish Malaise Trap Project (SMTP); the Biologiska Museet (MZLU), Lund and the Naturhistoriska Riksmuseet
(NHRS), Stockholm. In addition the private collections of Anders Nilsson, Johan Abenius, Sven Hellqvist, Bo G. Svensson and Niklas Johansson. The results were checked by the second author with specimens deposited in the Naturalis Biodiversity Center
(RMNH), Leiden and the Oberösterreichisches Landesmuseum, Biologiezentrum (BZL), Linz.

## Systematics

### Gasteruption assectator

Taxon classificationAnimaliaHymenopteraGasteruptiidae

(Linnaeus, 1758) sensu stricto

**Figures 1–3.**
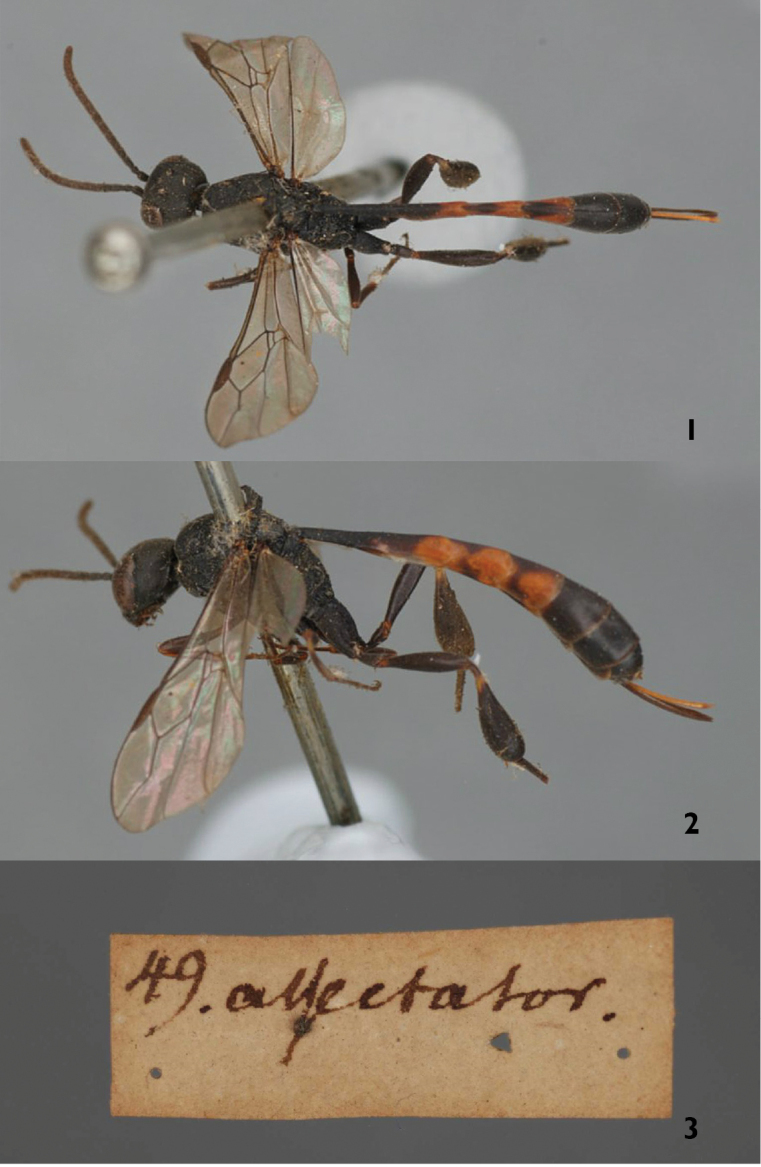
Lectotype of *Gasteruption assectator* (Linnaeus). **1** habitus dorsal **2** habitus lateral **3** labels.

Figs 1–3 , 25 , 26 , 28

Ichneumon assectator Linnaeus, 1758: 566, 1761: 407, 1767: 937; Scopoli 1763: 287; Fabricius 1775: 340, 1781: 435, 1787: 268; Gmelin 1790: 2696; Villers 1789: 174; Rossi 1790: 90; Christ 1791: 375; Petagna 1792: 365; Cederhjelm 1798: 163; Schrank 1802: 263; Hentschius 1804: 112; Illiger 1807: 74; Roman 1932: 2; Hedqvist 1973: 182; Fitton 1978: 376.
Foenus assectator ; Fabricius 1798: 240; Walckenaer 1802: 75; Latreille 1805: 195; Dahlbom 1831: 77; Curtis 1832: 423; Nees 1834: 308; Stephens 1835: 121; Labram and Imhoff 1836: 24; Zetterstedt 1840: 408; Westwood 1843: 255; Taschenberg 1866: 93; Tournier 1877: ix (as affectator); Thomson 1883: 849.
Foenus affectator ; Abeille de Perrin 1879: 265, 266, 277.
Gasteruption assectator ; Schletterer 1885: 276, 316, 1889: 384, 393, 395, 397; Dalla Torre 1902: 1063; Szépligeti 1903: 370 (as affectator); Kieffer 1912: 256 (id.); Lindemans 1921: 298 (id.); Roman 1932: 2; Schmiedeknecht 1930: 380, 383 (as affectator); Hedicke 1939: 5 (id.); Ferrière 1946: 235, 238, 240 (id.); Leclercq 1948: 75; Hellén 1950: 4; Townes 1950: 123–128; Šedivý 1958: 36, 37; Györfi and Bajári 1962: 48, 51; Schmidt 1969: 293; Hedqvist 1973: 181; Fitton 1978: 376; Dolfuss 1982: 22; Oehlke 1984: 169, 171, 175; Ortega and Baez 1985: 509, 515; Madl 1987a: 401, 1987b: 21, 1988: 37, 1989a: 159, 1989b: 41, 1990a: 127, 1990b: 480; Kozlov 1988: 245, 247; Kofler and Madl 1990: 320; Narolsky and Shcherbal 1991: 23, 24; Wall 1994: 150; Scaramozzino 1995: 3; Smith 1996: 492; Peeters 1996: 134; Neumayer et al. 1999: 220; Pagliano and Scaramozzino 2000: 11, 19; Saure 2001: 29; Yildirim et al. 2004: 1350; Turrisi 2004: 84; Westrich 2008: 7–8; van der Smissen 2010: 372; Zhao et al. 2012: 23–27; van Achterberg 2013: 82; van Achterberg and Talebi 2014: 57–61.
Gasteruption affectator ; Semenov 1892: 200.
Ichneumon annularis Geoffroy in Fourcroy 1785: 398; Hedicke 1939: 7; Wall 1994: 148 (type lost). Synonymized by with Gasteruption assectator (Linnaeus) by Olivier (1792).

#### Type material.

**Figures 4–7.**
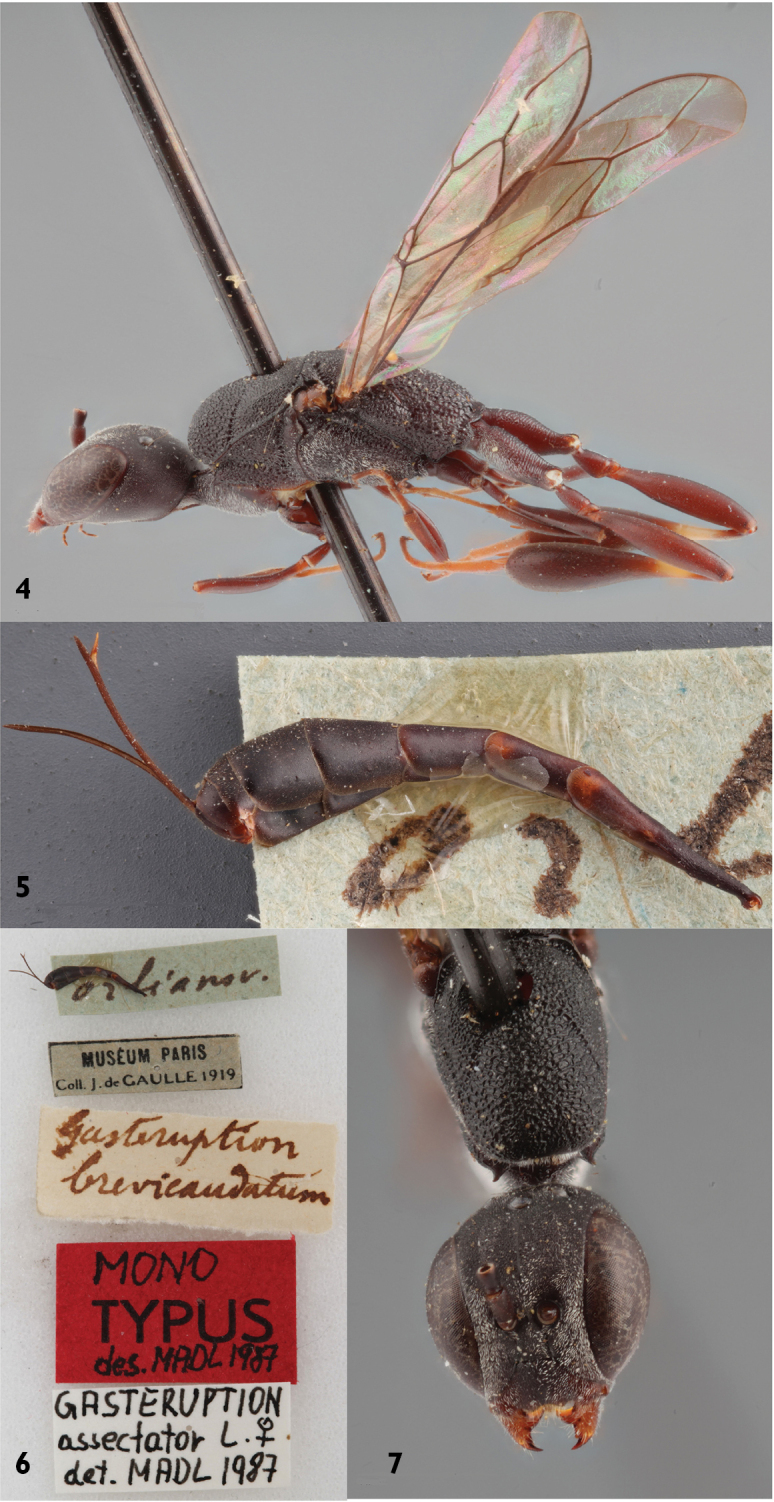
Lectotype of *Gasteruption brevicauda* (Kieffer). **4** habitus lateral **5** metasoma lateral **6** labels **7** mesoscutum and head dorsal.

High resolution photos of the lectotype female of *Gasteruption assectator* in the Linnaean collection coll. no 2652- “49 *assectator*” (Figs 1–3) designated by van Achterberg and Talebi (2014) was studied. The specimen has an unusually short ovipositor and the pilosity of the sheath is longer than average, but within the variation of the species. The holotype female of *Gasteruption brevicauda* (Figs 4–7) from Algeria (Orléansville) was examined and the specimen, with its strongly sculptured mesoscutum, the strong antero-lateral teeth of the pronotum aswell as the orange hind tarsus clearly belongs to *Gasteruption undulatum* (Abeille de Perrin, 1879). The synonymisation with *Gasteruption assectator* made by Madl (1987a) is here rejected and *Gasteruption brevicauda* Kieffer, 1904, is a new synonym of *Gasteruption undulatum* (Abeille de Perrin, 1879) syn. n.

#### Additional material.

**Sweden** (***Skåne***: Åhus, ***Blekinge***; ***Halland***: Breared; ***Småland***: Repperda, Bäckebo, Hälleskog, Tvärskog, Robacken, Igersdela, Skillingaryd, Södra Vi, Korsberga; ***Gotland***: Ardre, Stora Karlsö, Fårö, Mullvalds; ***Öland***: Halltorp, Ekerum, Glömminge; ***Östergötland***: Simonstorp, Borensberg)

#### Diagnosis.

Temples in dorsal view less parallel-sided and usually shorter than of *Gasteruption boreale*, head in dorsal view transverse, mostly distinctly wider than long. Occipital carina indistinct and not reflexed. Face mostly slightly narrower than that of *Gasteruption boreale*. Hypostomal bridge narrow, at most 0.5 times mandibular base (Fig. 25). Mesoscutum in most cases distinctly reticulate-coriaceous and without satin sheen (Fig. 26), medio-posteriorly in front of scutellum distinctly rugose. Mesosoma and head silvery pilose. Mesosomal surface with a fatty gloss, quite distinct from the more opaque satin sheen in *Gasteruption boreale*. Antenna slightly longer than in *Gasteruption boreale*, with sixth segment about 1.8 times longer than wide and subapical segment about 1.5 times longer than wide. Hind coxa dorsally striate-rugose. Hind tibia and basitarsus with white ring which might be interrupted ventrally. Metasoma mainly black with lateral orange patches on tergites 2–4 often merged. Fore and middle tibiae with small, but quite distinct white or yellow patch basally. Ovipositor sheath black or brown, 1.0–1.3 times as long as hind tibia and without prominent bristles but with thinner adpressed pubescence, appearing nearly naked (Fig. 28). The pilosity of equal intensity all over the surface not becoming scarcer towards the tip. In some specimens, especially when the sheath parts are twisted as in the lectotype female, the pilosity might be slightly raised. The species is closely related to *Gasteruption boreale* (Thomson, 1883) and *Gasteruption nigritarse* (Thomson, 1883), but the female can be distinguished by the slightly longer ovipositor without conspicuous bristles. The male is distinguishable by its slightly shorter head in dorsal view and the often more distinctly reticulate-rugose mesoscutum without satin sheen.

#### Distribution.

*Gasteruption assectator* is the most widespread and common species of the *assectator* aggregate in Europe. Towards its northern distribution limits in northern Scandinavia it seems to be confined to coastal areas with more favorable climate than inland areas.

#### Biology.

*Gasteruption assectator* occurs in a wide variety of habitats, varying from agricultural landscapes to deciduous forests and gardens. Most probably *Hylaeus* spp. are used as hosts.

### Gasteruption boreale

Taxon classificationAnimaliaHymenopteraGasteruptiidae

(Thomson, 1883) stat. rev.

Figs 8–11 , 12–13 , 14–15 , 16 , 17–18 , 27 , 29

Foenus borealis Thomson, 1883: 849; Hedicke 1939: 7; Hedqvist 1973: 181, 182 (invalid lectotype designation); Wall 1994: 148. Synonymized with Gasteruption assectator (Linnaeus) by Schletterer (1889) and with Gasteruption minutum (Tournier) by van Achterberg and Talebi (2014).
Gasteruption boreale ; Schletterer 1885: 303.
Foenus fumipennis Thomson, 1883: 848; Hedicke 1939: 7; Hedqvist 1973: 181, 182 (lectotype designation); Wall 1994: 148. Synonymized with Gasteruption assectator (Linnaeus) by Schletterer 1885. **Syn. n.**
Trichofoenus breviterebrae Watanabe, 1934: 285; Hedicke 1939: 45. Synonymized with Gasteruption assectator (Linnaeus) by Pagliano and Scaramozzino (2000). **Syn. n.**
Gasteruption margotae Madl, 1987c: 225–227, 1990b: 480; Wall 1994: 149. Synonymized with Gasteruption assectator (Linnaeus) by Madl (1990b). **Syn. n.**

#### Type material.

**Figures 8–11.**
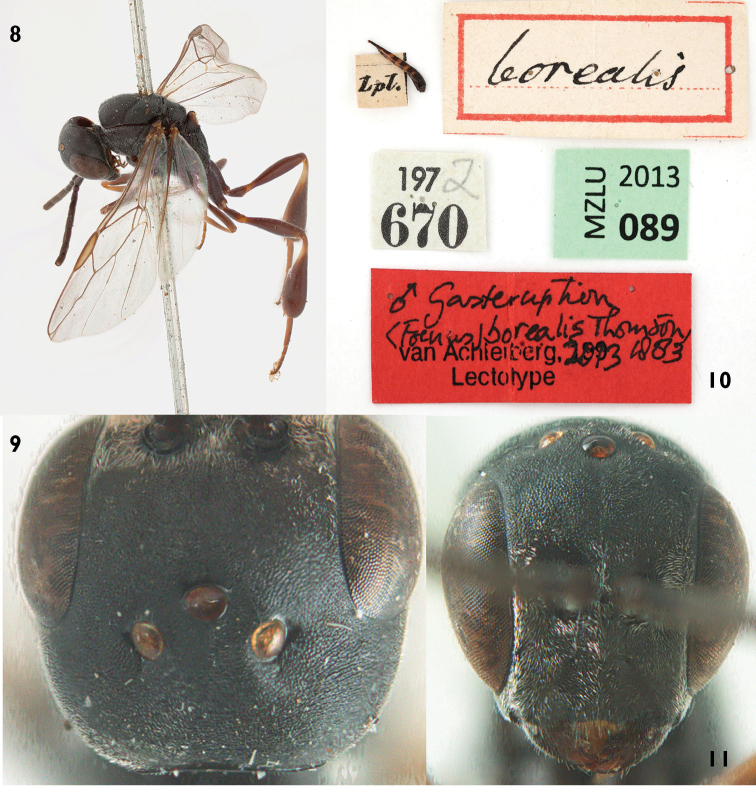
Lectotype of *Gasteruption boreale* (Thomson). **8** habitus lateral **9** head dorsal **10** labels **11** head anterior.

In Thomsons collection in MZLU four males and one female are placed at the label *Foenus borealis*. Hedqvist (1973) states that the type series by Thomson only consisted of three males and one female but this is probably a simple miscalculation. The female (from Norway) was originally selected as lectotype by Hedqvist (1973) but the selection was declared as invalid (van Achterberg and Talebi 2014) because the listed original locality of the type series (= Lappland) excludes the selection of a lectotype from Norway. One male (Figs 8–11) was designated lectotype by van Achterberg and Talebi (2014) and the species was synonymized with *Gasteruption minutum* (Tournier, 1877). All males and the female in the type series belong to one distinct species (see key below). The wider malar space exhibited by the lectotype is clearly shorter than the mandibular base and fits within the range of this species and is not as long as in *Gasteruption minutum*.

**Figures 12–13.**
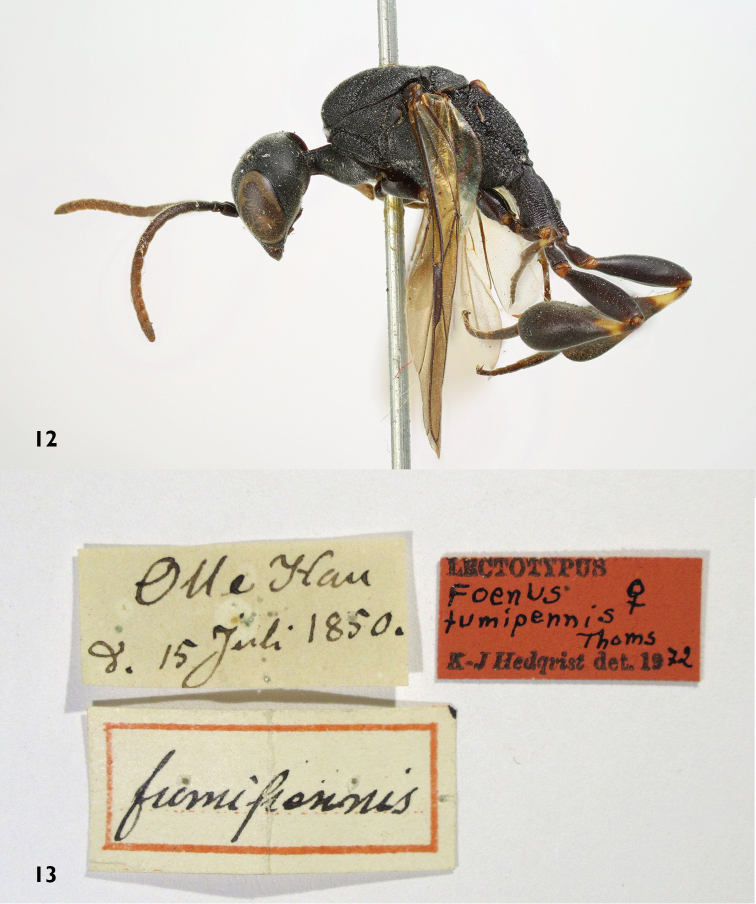
Lectotype *Foenus fumipennis* Thomson. **12** habitus lateral **13** labels.

The type series of *Gasteruption fumipenne* consists of the lectotype from Gotland. The size, habitus, antennae and smooth sculpture on the mesoscutum of the lectotype (Figs 12–13) indicates, despite the lacking metasoma, that it concerns a female of *Gasteruption boreale*. This is the only specimen known from the Baltic island Gotland, but the type locality (“Olle hau”= Ulla hau, Fårö, Gotland, Sweden) was at the time of the collection an active sand dune field with old pines. It was quite a different ecosystem than at the mainland of Gotland where *Gasteruption assectator* is the most common (and now only?) of the three species. The other specimen under this label is a male of *Gasteruption assectator* from Scania (Skåne). The synonymisation with *Gasteruption assectator* made by Schletterer (1889) is rejected and *Foenus fumipennis* is to be regarded as a new synonym of *Gasteruption boreale* (Thomson).

**Figures 14–15.**
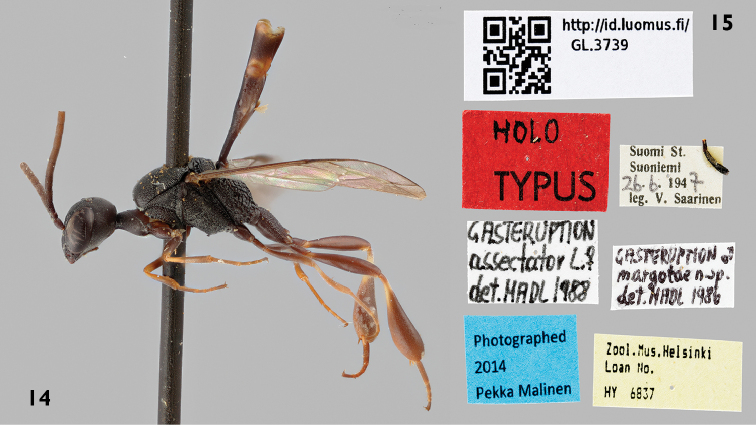
Holotype of *Gasteruption margotae* Madl. **14** habitus lateral **15** labels.

The type series of *Gasteruption margotae* consists of the male holotype (Figs 14–15) from Finland (Madl 1987c); the holotype is a typical male of *Gasteruption boreale* (Thomson). The study of the holotype shows that the synonymisation with *Gasteruption assectator* made by Madl (1990) is unjustified after resolving the *Gasteruption assectator* aggregate and that *Gasteruption margotae* is clearly a new synonym of *Gasteruption boreale* (Thomson).

**Figure 16.**
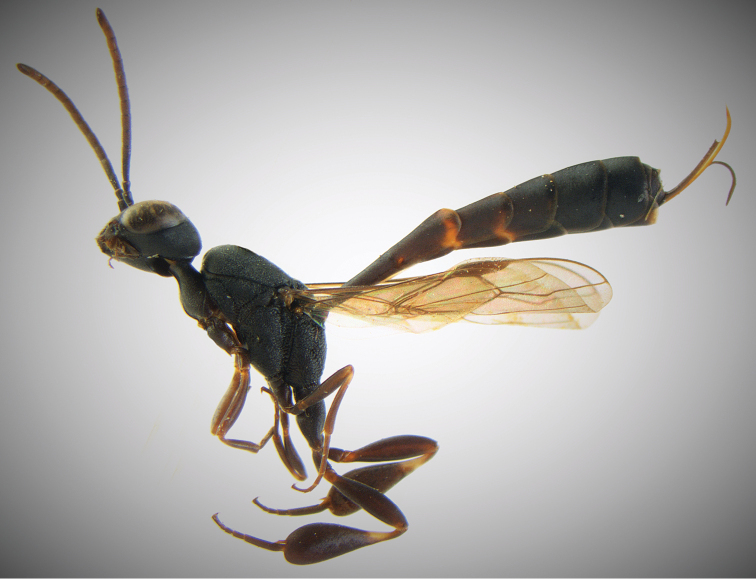
Lectotype of *Trichofoenus breviterebrae* Watanabe, habitus lateral.

The examined type series of *Gasteruption breviterebrae* (Fig. 16) consists of the holotype female and a paratype male from Sakhalin (Far East Russia). The holotype shows the typical features of *Gasteruption boreale* (Thomson), viz., the less sculptured mesoscutum and the bristly ovipositor sheath. The slightly aberrant red marks on the metasoma fall within the geographical variation of *Gasteruption boreale*. The synonymisation with *Gasteruption assectator* made by Pagliano and Scaramozzino (2000) is here rejected and *Trichofoenus breviterebrae* is a new synonym of *Gasteruption boreale* (Thomson).

#### Additional material.

**Sweden** (***Småland***: Bäckebo, Skillingaryd, Ränneslätt, Jönköping; ***Västergötland***: Baskarp; ***Södermanland***: Huddinge; ***Uppland***: Rossholm; ***Dalarna***: Leksand, Ludvika; **Hälsingland**: Hornslandet; ***Västerbotten***: Vindeln, Hällnäs; ***Lycksele lappmark***: Gällivare); **Finland** (***Åland***: Hammarland; ***Åbo***: Harvaluoto).

#### Diagnosis.

**Figures 17–18.**
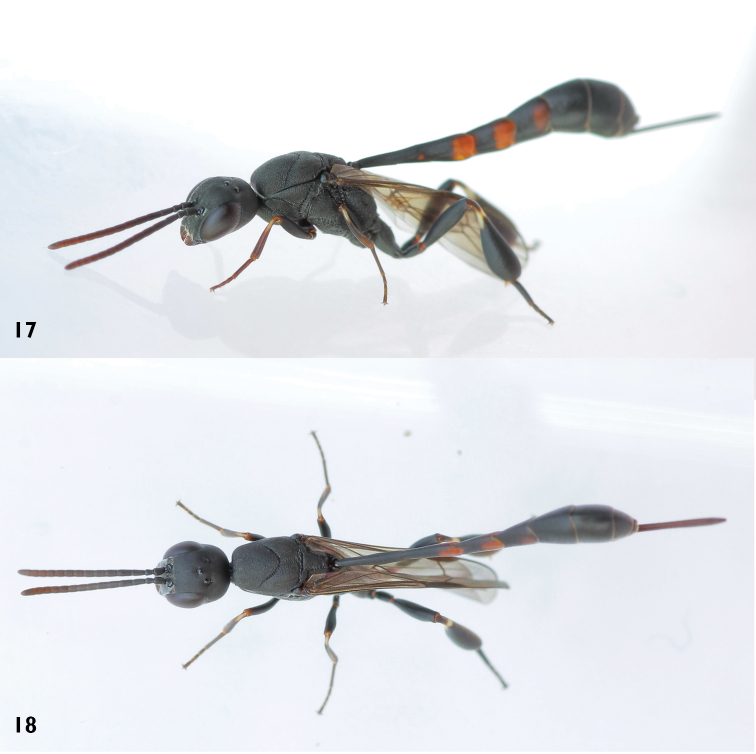
*Gasteruption boreale* (Thomson), ♀. **17** habitus lateral **18** habitus dorsal.

Head in dorsal view almost parallel-sided behind eyes, elongate, about as wide as long (Figs 9, 18). Occipital carina indistinct and not reflexed. Frons with satin sheen. Mesoscutum smooth, weakly rugose/shagreened with satin sheen, medio-posteriorly in front of scutellum rugose-reticulate (Figs 17–18, 27). Mesosoma and head silvery pilose. Mesosoma with a satin sheen, quite distinct from the rather matt gloss occurring in *Gasteruption assectator*. Whitish pubescence of eye of female mostly distinctly longer and denser than of *Gasteruption assectator*. Antenna slightly shorter than in *Gasteruption assectator* with sixth segment about 1.5 times longer than wide and subapical segment about 1.2 times longer than wide. Only apical half of hind coxa weakly striate dorsally. Hind tibia and basitarsus with white ring which might be interrupted ventrally. Metasoma mainly black with orange lateral patches on tergites 2–4 which might be partially reduced, especially in northern specimens. Inner sides of tibiae often red brown to orange with white or yellow basal patch indistinct on fore and middle tibiae. Ovipositor sheath black or brown, 0.7–1.0 times as long as hind tibia, its apical half entirely with stout, rather scarce black bristles angled backwards at about 45° (Fig. 29). The species is closely related to *Gasteruption assectator* (Linnaeus) but the female can be distinguished by the shorter ovipositor sheath, the less sculptured mesoscutum and the more scarce prominent bristles on the apical half of the ovipositor sheath. The male is hard to distinguish from *Gasteruption assectator* and identification is not always possible with certainty. In most cases the male of *Gasteruption boreale* can however be separated from *Gasteruption assectator* by its slightly more elongated, parallel-sided head in dorsal view, the more or less enlarged malar space and its less sculptured mesoscutum.

#### Description.

Female. Length of body 6–11 mm (fore wing 3.5–5.5 mm)

*Head*. Temples parallel-sided behind eyes in dorsal view. Occipital carina not raised. Frons and vertex with satin sheen. Malar space short, at most about 0.5 times mandibular base. Hypostomal bridge narrow, at most 0.5 times mandibular base. Eyes with dense white pubescence. Antenna short; sixth segment about 1.5 times longer than wide and subapical segment about 1.2 times longer than wide.

*Mesosoma*. Surface largely smooth with satin sheen, mesoscutal sculpture of almost equal intensity as on vertex. Antesternal carina narrow, non-lamelliform. Pronotal sides with very small pointed teeth antero-ventrally, but these are sometimes entirely absent. Upper half of mesopleuron mostly considerably weaker sculptured than its more rugose lower part.

*Legs*. Hind tibia stout as in *Gasteruption assectator*. Hind coxa often with weaker rugae apically than on basal half, dissolving amidst rugose background. Hind tibial spurs often brighter than hind tibia.

*Metasoma*. Ovipositor sheath entirely black or brown, 0.7–1.0 times as long as hind tibia, its apical half entirely with stout, black bristles angled backwards at about 45° (Fig. 29).

*Colour*. Black. Mandible apically, hind tibial spurs and patches laterally on tergites 2–4 reddish brown. Patches rarely intercepted. Fore and middle tibiae mostly with indistinct yellow or ivory basal patch. Inner side of tibiae often orange. Basal ivory ring of hind tibia usually indistinct.

#### Distribution.

As Thomson’s name implies this species is most common in boreo-alpine areas. In northern Europe it is quite widespread and common at higher latitudes and high altitude sites in the southern part, but becoming scarcer towards the southern lowlands in Sweden. Specimens are examined from Austria, Bulgaria, Finland, Germany, Netherlands (den Dolder, de Bilt, Tilburg, Wageningen, Groesbeek, Rhenen, Velp, Maastricht, Drimmelen, Rotterdam, Voerendaal), Norway, Russia, Serbia and Sweden.

#### Biology.

The species occur in (boreal) landscapes dominated by coniferous forests where it can be locally common. Many of the sites in Scandinavia are at high altitude. *Gasteruption boreale* is lacking from more open localities as well as in regions dominated by deciduous forests. It has been observed searching high stumps of *Pinus* trees and it also attends old wooden walls and artificial bee nests in gardens. Probably it is a kleptoparasitoid of *Hylaeus* spp.

### Gasteruption nigritarse

Taxon classificationAnimaliaHymenopteraGasteruptiidae

(Thomson, 1883) stat. rev.

**Figures 19–22.**
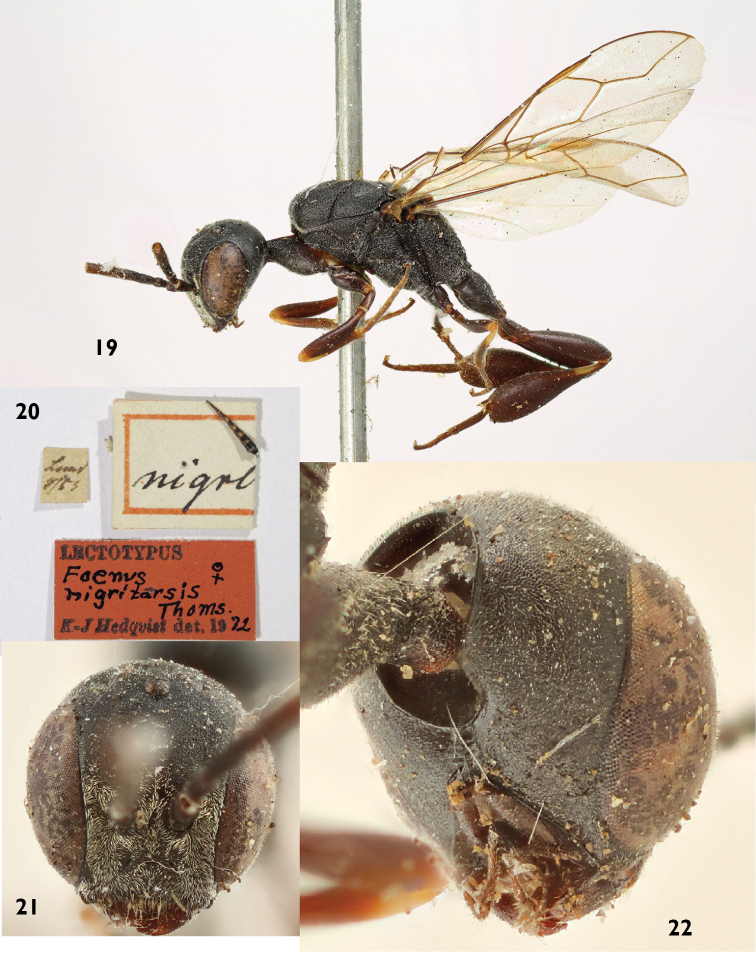
Lectotype of *Gasteruption nigritarse* (Thomson). **19** habitus lateral **20** labels **21** head anterior **22** head ventral.

**Figure 23.**
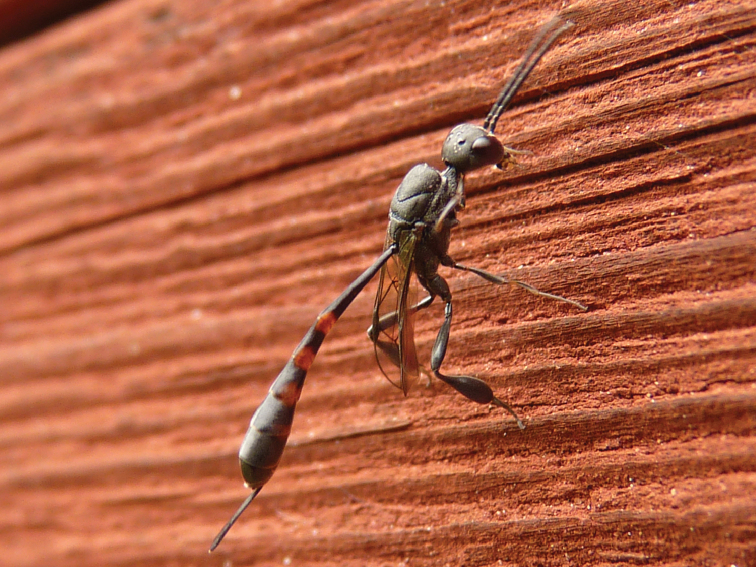
*Gasteruption nigritarse* (Thomson), ♀, habitus dorso-lateral.

**Figures 24–25.**
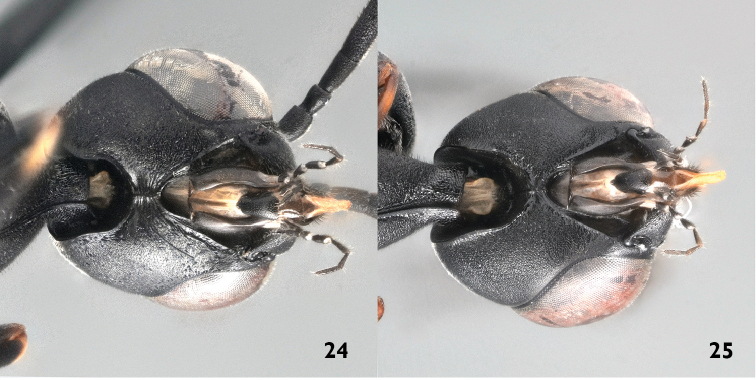
Hypostomal bridge of *Gasteruption nigritarse* (Thomson) (**24**) and *Gasteruption assectator* (Linnaeus) (**25**).

Figs 19–22 , 23 , 24 , 30

Foenus nigritarsis Thomson, 1883: 849; Schletterer 1889: 398; Hedicke 1939: 7; Hedqvist 1973: 181, 182 (lectotype designation); Wall 1994: 149. Synonymized with Gasteruption assectator (Linnaeus) by Schletterer (1889).
Gasteruption nigritarse ; Schletterer 1885: 310.

#### Type material.

Lectotype female (Figs 19–22) from Lund (Scania) selected by Hedqvist (1973). All 12 specimens under *Foenus nigritarsis*, both males and females (including the designated lectotype by Hedqvist) belong to the same distinct species and are well separable from *Gasteruption assectator* *sensu stricto* (see key below).

#### Additional material.

**Sweden** (***Småland***: Bäckebo; ***Skåne***; ***Halland***; ***Östergötland***: Svensksund; ***Uppland***: Grisslehamn, Svartsjö, Roslagsbro, Skansen; ***Öland***: Borgehage, Himmelsberga).

#### Diagnosis.

Head dorsally more parallel-sided than in *Gasteruption assectator*, elongate and about as wide as long. Occipital carina indistinct and not reflexed. Mesoscutum superficially reticulate and (especially laterally) rugose, medio-posteriorly in front of scutellum more rugose-reticulate. Mesosoma laterally and face with long, thick golden pubescence. Hind tibia and basitarsus darker, often with the basal ring lacking or interrupted. Fore and middle tibiae often with large distinct ivory patch covering about one third of tibia. Metasoma mainly black with well-defined orange lateral patches on tergites 2–5 which might be partially reduced, especially in northern specimens. Ovipositor sheath entirely black or brown, 0.7–1.0 times as long as hind tibia and its apical half dorsally with stout, black bristles angled backwards at about 45°. The bristles are all conspicuously widened and bent apically, reminiscent of “velcro” (Fig. 30). The species is closely related to *Gasteruption assectator* (Linnaeus) and *Gasteruption boreale* (Thomson), but the female can be distinguished by the stout velcro-like bristles dorsally on the apical half of the ovipositor sheath, its denser pubescence of head and mesosoma and its broader hypostomal bridge. The male is distinguishable by its broader hypostomal bridge as well as the thick golden facial pubescence.

#### Description.

Female. Length of body 8–11 mm (fore wing 4.0–5.5 mm)

*Head*. Temples parallel-sided behind eyes in dorsal view. Occipital carina not raised. Frons and vertex with satin sheen. Malar space short, at most about 0.5 times mandibular base. Hypostomal bridge at least 0.7 times width of mandibular base, medio-laterally often with distinct transverse striae. Face covered with dense golden pubescence.

*Mesosoma*. Surface vaguely reticulate and strongly shagreened. Antesternal carina narrow and non-lamelliform. Pronotal sides with very small pointed teeth antero-ventrally, but these are sometimes entirely absent.

*Legs*. Hind tibia rather stout as in *Gasteruption assectator*. Hind tibial spurs and hind tibia mostly similarly coloured.

*Metasoma*. Ovipositor sheath entirely black or brown, 0.7–1.0 times as long as hind tibia, its apical half dorsally with stout, black bristles angled backwards at about 45° and conspicuously widened and bent backwards apically.

*Colour*. Black. Mandible apically orange. Sometimes small patch on hind tibia baso-ventrally white or ivory. Northern specimens of both sexes often with entirely black hind tibia. Fore and middle tibiae often with large distinct ivory patch covering about one third of tibia. Fore, middle and hind tarsus black. Patches laterally on tergites 2–5 reddish brown. Last sternite apically often extensively orange. Colour of hind tibial spurs variable, mostly black or dark brown.

#### Distribution.

*Gasteruption nigritarse* is a rather rare locally but widespread species in Europe. Specimens examined from Austria, Germany, Czech Republic, Netherlands (Breda, Waalwijk, Putten (GE), Maastricht, Wageningen, Rhenen, Arnhem), Serbia, Sweden and Turkey.

#### Biology.

The species primarily occur in small-scale agricultural landscapes where it is to be found especially on walls of log barns (Fig. 23). An association with the bees *Hylaeus difformis* and/or *Hylaeus pictipes* is highly probable, at least in Scandinavia and is based on observed behaviour of the wasps. *Gasteruption nigritarse* seems to have diminished dramatically in Scandinavia during the last century, probably due to the loss of habitat and is only known from a couple of localities.

### Key to Palaearctic species of the *Gasteruption assectator* aggregate

**Table:** 

1	Females (ovipositor present)	**2**
–	Males (ovipositor absent)	**4**
2	Hypostomal bridge 0.7–0.8 times as wide as mandibular base and weakly striate medio-laterally (Figs 22, 24). Occipital carina conspicuously bent inwards medio-ventrally, resulting in parallel running lower parts (Figs 22, 24). Hind tibia in northern populations often with reduced white markings. In lateral view apical half of ovipositor sheath with hooked bristles (“velcro-type”; Fig. 30). Facial pubescence thick and golden	***Gasteruption nigritarse* (Thomson, 1883), stat. rev.**
–	Hypostomal bridge narrow, at most 0.5 times as wide as mandibular base and without striation medio-laterally (Fig. 25). Occipital carina evenly diverging medio-ventrally (Fig. 25). Hind tibia mostly with distinct white ring basally. Ovipositor sheath without “velcro”-type of bristles (Figs 28–29). Facial pubescence thin and silvery	**3**
3	Mesoscutum and coxae more opaque, less rugose, smoother and with satin sheen (Figs 17–18, 27). Sculpture of mesoscutum and head similar, shagreened (Fig. 27). Ovipositor sheath 0.7–1.0 times as long as hind tibia, in dorsal view its apical half with more scarse distinct straight bristles, angled backwards at about 45° (Fig. 29)	***Gasteruption boreale* (Thomson, 1883), stat. rev.**
–	Mesoscutum and coxae more shiny and rugose, with a “fatty” gloss (Fig. 26). Sculpture of mesoscutum distinctly rougher than that of head, reticulate-coriaceous (Fig. 26). Ovipositor sheath 1.0–1.3 times as long as hind tibia, in dorsal view normally only with dense bright adpressed pubescence, thus appearing naked in lower magnifications (Fig. 28)	***Gasteruption assectator* (Linnaeus, 1758)**
4	Hypostomal bridge 0.7–0.8 times as wide as width of mandibular base and weakly striate medio-laterally (Figs 22, 24). Occipital carina conspicuously bent inwards medio-ventrally, resulting in parallel running lower parts (Figs 22, 24). Hind tibia in northern populations often with reduced white markings. Facial pubescence thick and golden	***Gasteruption nigritarse* (Thomson, 1883), stat. rev.**
–	Hypostomal bridge narrow, at most 0.5 times width of mandibular base and without striation medio-laterally (Fig. 25). Occipital carina medio-ventrally evenly diverging. Hind tibia mostly with distinct white ring basally. Facial pubescence thin and silvery	**5**
5	Mesoscutum and upper half of mesopleuron rather smooth with small puncture-like grooves and with satin sheen almost of the same intensity as vertex (Figs 17–18, 27). Head, mesosoma and coxae more opaque and with satin sheen. Sculpture of mesoscutum similar to that of head (Fig. 27). Head in dorsal view more elongated and its temples more parallel-sided behind eyes (Fig. 10)	***Gasteruption boreale* (Thomson 1883), stat. rev.**
–	Mesoscutum more roughly reticulate-coriaceus, especially near the sides of mesoscutum, visible even at lower magnifications (Fig. 26); mesoscutal sculpture distinctly different from the less rugose surface of vertex. Head, mesosoma and coxae with “fatty” gloss. Head in dorsal view less elongated and clearly wider than long, generally more converging behind eyes	***Gasteruption assectator* (Linnaeus, 1758)**

**Figures 26–27.**
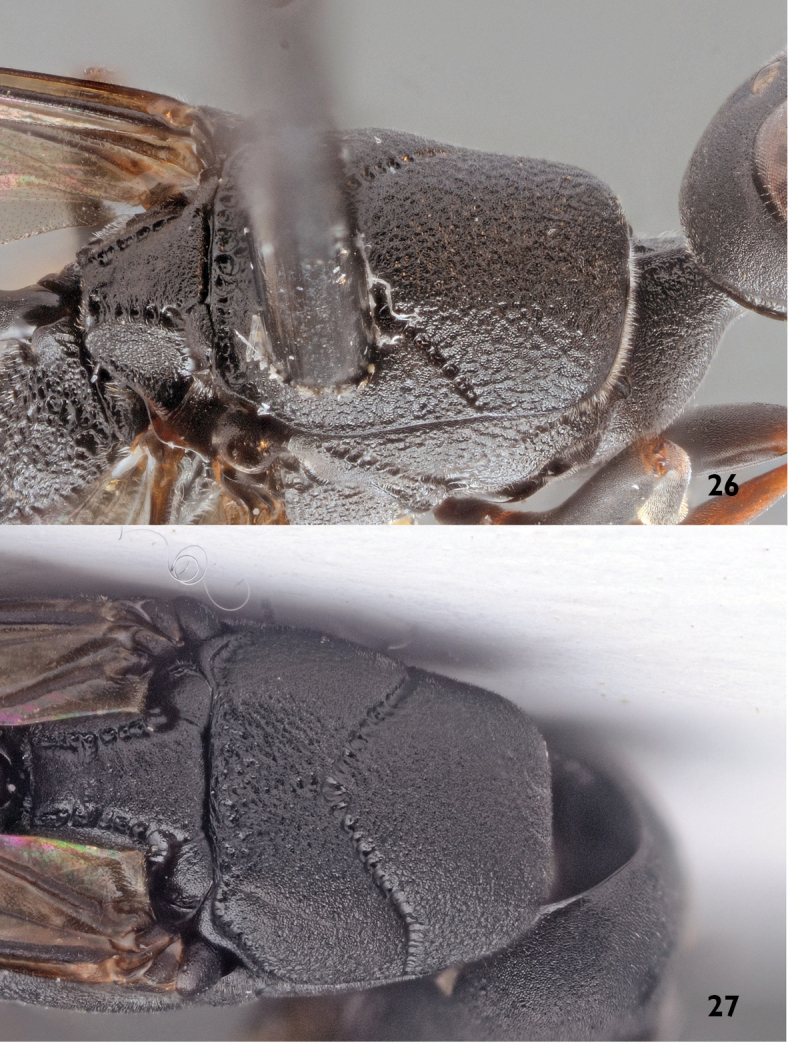
Mesoscutum of *Gasteruption assectator* (Linnaeus) (**26**) and *Gasteruption boreale* (Thomson) (**27**).

**Figures 28–30.**
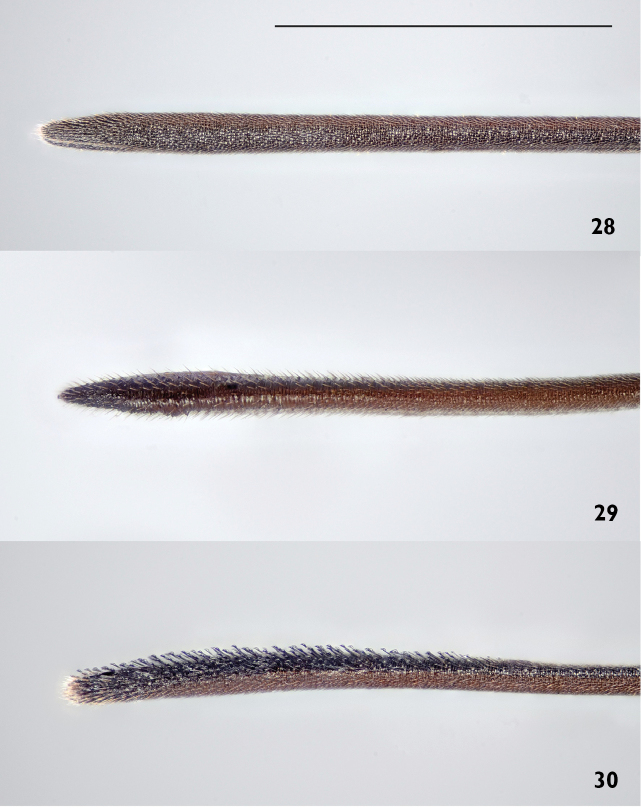
Ovipositor sheath of *Gasteruption assectator* (Linnaeus (**28**), *Gasteruption boreale* (**29**) and *Gasteruption nigritarse* (Thomson) (**30**). Scale bar 1 mm.

## Supplementary Material

XML Treatment for Gasteruption assectator

XML Treatment for Gasteruption boreale

XML Treatment for Gasteruption nigritarse
